# Association of circulating let-7b-5p with major depressive disorder: a nested case-control study

**DOI:** 10.1186/s12888-021-03621-4

**Published:** 2021-12-09

**Authors:** Sanne Roumans, Kristina Sundquist, Ashfaque A. Memon, Anna Hedelius, Jan Sundquist, Xiao Wang

**Affiliations:** 1 Center for Primary Health Care Research, Department of Clinical Sciences Malmö, Lund University, Lund, Sweden; 2 Department of Family Medicine and Community Health, Department of Population Health Science and Policy, Icahn School of Medicine at Mount Sinai, New York, USA; 3 Center for Community-based Healthcare Research and Education (CoHRE), Department of Functional Pathology, School of Medicine, Shimane University, Matsue, Japan; 4 Center for Primary Health Care Research, Wallenberg Laboratory, Inga-Marie Nilssons gata 53, plan 6 Box 50332, 202 13 Malmö, Sweden

**Keywords:** Let-7b-5p, Major depressive disorder, MDD, Depression, miRNA, Biomarker, Risk prediction

## Abstract

**Background:**

Major depressive disorder (MDD) is one of the most common psychiatric disorders and is a great disease burden. However, its underlying pathophysiology and aetiology remain poorly understood. Available evidence suggests that circulating microRNAs (miRNAs) are associated with MDD, but it is still unknown whether miRNAs can predict subsequent incident MDD.

**Methods:**

In this nested case-control study, a total of 104 individuals, who were free of MDD at baseline, from the Women’s Health in Lund Area (WHILA) cohort were included. Among them, 52 individuals developed MDD (cases) during the 5 years follow-up and 52 individuals did not develop MDD (controls). Plasma expression levels of miR-17-5p, miR-134-5p, miR-144-5p, let-7b-5p and let-7c-5p at baseline were assessed using qRT-PCR. Logistic regression was used to estimate the odds of developing MDD among individuals with different levels of miRNA expression.

**Results:**

Plasma expression levels of let-7b-5p were significantly lower (*p* = 0.02) at baseline in cases compared to controls. After adjustment for age and BMI, let-7b-5p was negatively associated with odds for developing MDD (OR = 0.33, *p* = 0.03, 95% CI = 0.12–0.91). Moreover, let-7b-5p expression levels showed a trend over time with larger differences between cases and controls for the earlier cases (MDD diagnosis <2 years from baseline) than MDD cases developed later (MDD diagnosis 2–5 years from baseline).

**Conclusions:**

These findings show that lower plasma levels of let-7b-5p are associated with a higher future risk of MDD. Results need to be validated in a large cohort to examine its potential as a peripheral biomarker for MDD.

**Supplementary Information:**

The online version contains supplementary material available at 10.1186/s12888-021-03621-4.

## Background

Major depressive disorder (MDD) is a medical condition that includes abnormalities in mood, cognition, appetite, sleep, and psychomotor activity [1]. MDD is one of the most common psychiatric disorders worldwide and is currently projected to become the condition with the greatest disease burden after cardiovascular disease (CVD) by 2030 [2]. As of today, clinical suspicion is still the key for the diagnosis of MDD [3] as the underlying pathophysiology and aetiology of depression remain poorly understood [4].

Previous research concerning the pathophysiology of depression has mainly focused on genetic factors that affect the risk of development of depression. However, few genes were found to affect the risk of development of depression [5]. More recently, the attention has shifted to epigenetics, as it has now been generally accepted that depression is a condition caused not only by genetic factors, but also by the influence of environmental factors on gene expression. Epigenetic mechanisms, including histone modifications, DNA methylation, and a newly recognised group of regulators, the non-coding RNAs (ncRNAs), contribute to the regulation of gene expression [6].

The most common ncRNAs are the microRNAs (miRNAs). MiRNAs are a class of endogenous non-coding single-stranded RNAs of approximately 21–23 nucleotides in length. MiRNAs are transcribed in the nucleus and transported to the cytoplasm where they can inhibit gene expression by either promoting messenger RNA (mRNA) degradation or by inhibiting translation through targeting of the 3′ untranslated region (UTR) of the target mRNA (Bartel [7]). MiRNAs are not only present and active in cells, but they are also observed in a stable, cell-free form. A number of studies have detected miRNAs in peripheral bodily fluids such as the whole blood, plasma, and cerebrospinal fluid (CSF) [8].

Over the past decade, circulating miRNAs have already been identified as potential biomarkers in other diseases such as cancer and diabetes [9–12]. In MDD, some miRNAs have been evaluated in multiple cohorts. MiR-17-5p was found to be significantly upregulated in patients with MDD compared to healthy controls (HC) [13, 14], whereas the expression of miR-134-5p, miR-144-5p, let-7b-5p and let-7c-5p was significantly downregulated [15–17].

These results indeed indicate a difference in miRNA expression levels between patients and healthy controls, but they do not clarify whether they are the underlying cause or the consequence of MDD. Biomarkers, which are the consequence of MDD, can be a clear indicator of an MDD diagnosis. Instead, biomarkers that are the cause of MDD might be used as a predictive tool for the risk prediction of the development of MDD, as well as a therapeutic target for the treatment of MDD.

The present study builds on the results of the aforementioned studies to determine whether a couple of miRNAs have the ability to predict the development of MDD. To our knowledge, this is the first study to investigate the prospective potential of miRNA expression in MDD. The study aimed to examine the association between miRNAs and the risk of MDD through the analysis of the expression levels (at baseline) of previously identified miRNAs (miR-17-5p, miR-134-5p, miR-144-5p, let-7b-5p and let-7c-5p) in a nested case-control study.

## Methods

### Sample selection

**Fig. 1:**
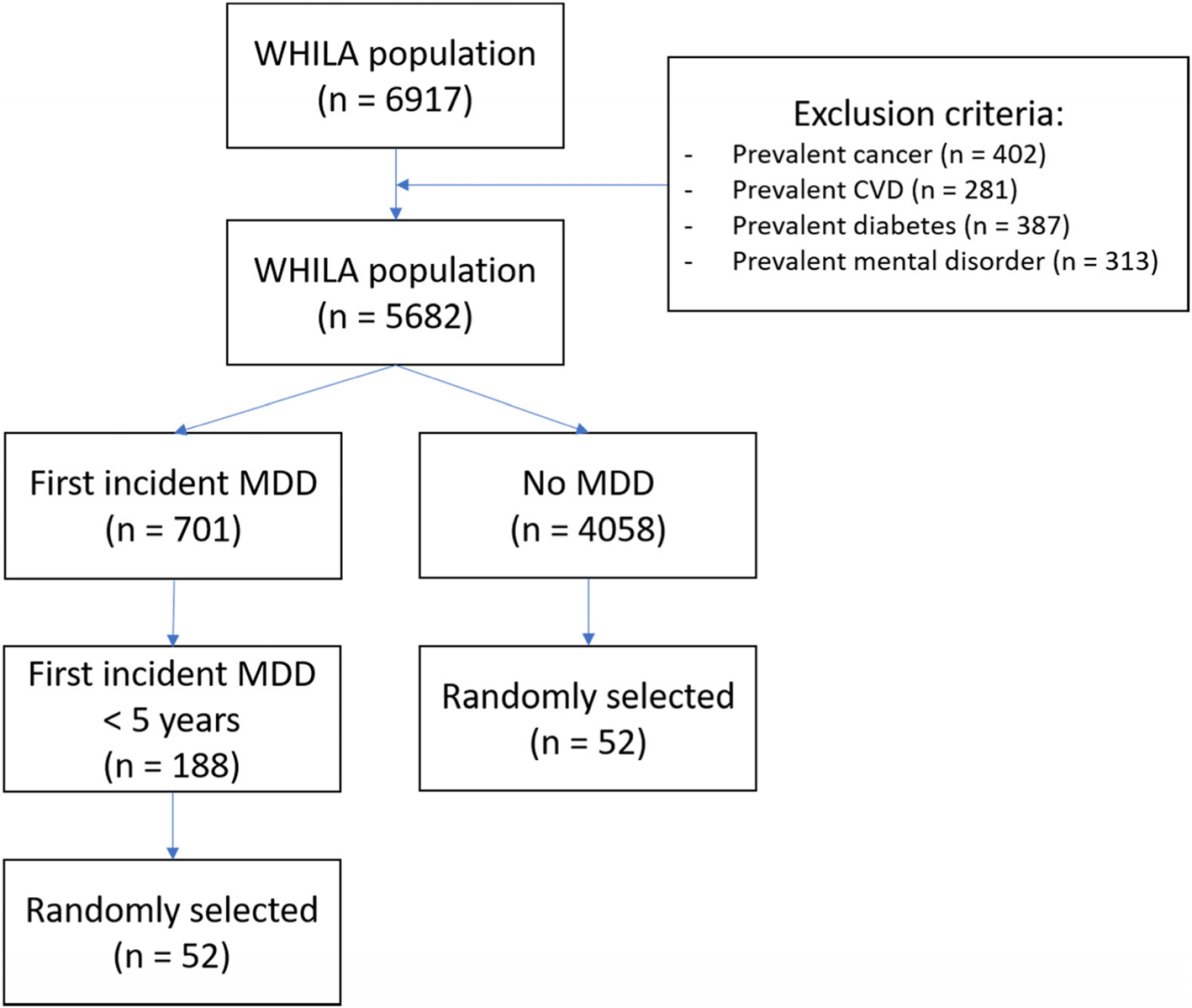
Flow chart of sample selection

Study subjects were selected from the Women’s Health in Lund Area (WHILA) cohort (Fig. 1); a cohort extensively described in a previous review [18]. Clinical data from the WHILA study were maintained at the Center for Primary Health Care Research (CPF). All women aged 50–65 years on December 1, 1995 in Lund, Skåne (*n* = 10,766) were invited to have their health status assessed. Participating women were included in the study from December 1, 1995, to February 3, 2000. A follow-up register study of the health care registers (hospital and out-patient) at the Social Welfare Board on all individuals in the WHILA study (*n* = 6917) was performed until May 31, 2015. A total of 818 individuals were diagnosed with MDD. The diagnoses of MDD in WHILA were selected from the registers and based on the International Classification of Diseases with ICD-codes (ICD7-ICD10): F32–33, 296B, 298A, 311, 302, 296.00, 298.00.

We excluded patients with prevalent cancer, prevalent CVD, prevalent diabetes (Type I and Type II), and prevalent mental disorder because these comorbidities were reported to be associated with the miRNAs selected in the present study [19–21].

Cases were randomly selected from subjects with the first diagnosis of MDD within 5 years after the baseline (*n* = 188), and controls were randomly selected from subjects without MDD or other mental disorders during follow-up (*n* = 4058). It was reported previously that miRNAs seem to be more closely associated with early events (diseases occurring 0–5 years from blood sampling) compared to later events (diseases occurring 6–10 years from blood sampling) [9]. Therefore, we selected individuals who developed MDD within 5 years.

Prior to the study, we performed a power calculation using previous results based on the expression of miR-144-5p [16]. This resulted in a sample size of 25 individuals in each group assuming 80% power and a significance level of 5%. Considering a possible skewed distribution of miRNA expression levels and missing samples, 52 cases of incident MDD during follow-up and 52 controls without MDD were included in the study.

### miRNA analysis

Plasma samples were collected at baseline as described in a previous paper [16]. Total RNA was isolated from 250 μL of each plasma sample using the miRNeasy Mini Kit (Qiagen, Hilden, Germany) according to the manufacturer’s protocol with minor adjustments. MS2 RNA was added to function as carrier RNA, while Sp2,4,5 RNA (spike-in RNA) was added as an internal control for RNA extraction efficiency. Four microlitre of isolated total RNA was reverse transcribed using the Universal cDNA Synthesis Kit (Exiqon, Aarhus, Denmark) according to the manufacturer’s protocol. Sp6 RNA (spike-in RNA) was added as an internal control for cDNA synthesis efficiency. qRT-PCR was performed with the miRCURY LNA™ SYBR Green PCR Kit (Qiagen, Hilden, Germany) according to the manufacturer’s protocol with minor adjustments in a Bio-Rad CFX384 Real-Time PCR Detection System. The following miRNA LNA™ primers (Qiagen, Hilden, Germany) were used: UniSp4 (cat. no. YP00203953), UniSp6 (cat. no. YP00203954), miR-425-5p (cat. no. YP00204337), miR-23a-3p (cat. no. YP00204772), miR-451a (cat. no. YP02119305), let-7b-5p (cat. no. YP00204750), let-7c-5p (cat. no. YP00204767), miR-134-5p (cat. no. YP00205989), miR-144-5p (cat. no. YP00204970), and miR-17-5p (cat. no. YP02119304). MiRNAs were tested in duplicate. Undetectable data were assigned a default threshold cycle value (Ct) of 37. MiR-451a and miR-23a-3p were used to detect haemolysis in the plasma samples [22]. Ct values were normalised according to the ∆Ct method with internal control miR-425-5p using the following equation: ∆Ct = Ct_miR-425-5p_ - Ct _miR of interest_. Normalisation stability of miR-425-5p was assessed in a previous study [16].

### Quality control

To ensure reproducibility of the samples, spike-in RNA was added during the RNA extraction and the cDNA synthesis. Both processes were assessed as reproducible as they had a coefficient of variation of 2.0 and 0.8%, respectively.

The quality of plasma samples was assessed through the measurement of haemolysis. Samples were considered to have haemolysis when the difference in Ct-value between miR-23a-3p and miR-451a was bigger than 7.0 [22]. Three samples were found to have haemolysis (∆Ct > 7.0) and were excluded in further analysis. All miRNAs were suitable for further analysis as less than 20% of Ct-values were > 37. MiR-134-5p had 17.2% of Ct-values >37.

### Ethical statement

This study was performed in-line with the principles of the Declaration of Helsinki. Sample storage and handling procedures were approved by the Lund/Malmö ethics committee (2011/494, 2015/6). Written informed consent was obtained from all subjects.

### Statistical analysis

Characteristic variables for all individuals were presented. Data were presented as the mean and standard deviation (SD) for age and body mass index (BMI) whereas smoking and alcohol use were presented as a percentage. Both parametric and non-parametric tests were performed to test the difference in miRNA expression levels at baseline between case and control samples. Pearson’s correlation test was used to examine the correlation between miRNAs and other available peripheral blood biomarkers that we had measured. The results of the Student’s t-test were used for further analysis of normally distributed miRNAs - miR-134-5p, miR-144-5p, and let-7b-5p - whereas results of the Wilcoxon rank-sum test were used for miRNAs with a skewed distribution. Logistic regression was used to examine the association between cases and controls for miRNA expression levels at baseline. We adjusted for age and BMI. Trends in miRNA expression levels over time were assessed via trend analysis of groups dichotomised over time until MDD. Results were deemed significant when *p* < 0.05. All statistical analyses were performed in STATA version 16 (StataCorp LP).

## Results

### Clinical characteristics

Clinical characteristics of both cases and controls at baseline are shown in Table 1. The mean age of the included women was 56 years, and the mean BMI was 24.51 kg/m^2^. Most women were non-smokers or low consumers of alcohol. There was no statistical difference between cases and controls in any of the confounders.

**Table 1:** Characteristics of the study population

Variable	Total (*n* = 104)	Case (*n* = 52)	Control (*n* = 52)	*p*-value
Age, years Mean (SD)	56.0 (2.78)	55.7 (2.57)	56.3 (2.97)	0.28^a^
BMI (kg/m^2^) ^c^ Mean (SD)	24.51 (3.92)	24.96 (3.86)	24.04 (3.98)	0.26^a^
Smoking (%) ^d^	25.7	25.5	26.0	0.95^b^
Alcohol abuse (%)	19.2	17.3	21.2	0.62^b^

Data are represented as mean (SD)

*SD* standard deviation

^a^Student’s t-test

^b^Two-sided Chi-squared test

^c^Missing information on BMI for 11 individuals

^d^Missing information on smoking for 3 individuals

### Comparison of baseline miRNA expression levels in cases and controls

During further analysis, a total of five samples were excluded due to poor quality of RNA, resulting in an analysis of 50 cases and 49 controls. MiRNA expression levels were measured for the five selected miRNAs. As shown in Table 2, let-7b-5p levels were significantly lower in MDD cases compared to controls (*p* = 0.02). For other miRNAs, significance was not reached but a trend of lower levels at baseline in cases compared to controls could be observed for miR-134-5p, miR-144-5p, and let-7c-5p. Levels at baseline in cases were higher for miR-17-5p than in controls, but not significant.

**Table 2:** Differentially expressed miRNAs (∆Ct) * in cases and controls at baseline

miRNAs	Case (*n* = 50)^a^	Control (*n* = 49)^a^	*p*-value
miR-17-5p	1.96 (0.38)	1.89 (0.32)	0.28^c^
miR-134-5p	−4.12 (1.91)	−4.07 (1.91)	0.82^b^
miR-144-5p	−2.99 (0.99)	−2.51 (1.56)	0.13^b^
let-7b-5p	1.53 (0.36)	1.59 (0.55)	0.02^b^
let-7c-5p	−0.08 (0.92)	0.06 (0.94)	0.37^c^

*IQR* interquartile range

^*^∆Ct = Ct_miR-425-5p_ – Ct_miR of interest_

^a^Median (IQR)

^b^Student’s t-test with equal variance

^c^Wilcoxon rank-sum test

Correlations between let-7b-5p and other peripheral blood biomarkers such as triglycerides (TG), total cholesterol (TC), high-density lipoprotein cholesterol (HDL-C), low-density lipoprotein cholesterol (LDL-C) and glucose were also analyzed. No correlations between let-7b-5p and the peripheral blood biomarkers were found (Table S1).

Logistic regression was used to estimate the odds ratio for having MDD among individuals with different levels of the selected miRNAs (Table 3). The results showed that let-7b-5p was negatively associated with the odds for having MDD (OR = 0.33, *p* = 0.03, 95% CI = 0.12–0.91). The results remained the same after adjustment for age and BMI.

**Table 3:** Risk estimates for MDD development based on single miRNA levels (∆Ct)

miRNAs*	Univariate analysis			Multivariate analysis		
	OR	***p***-value^**a**^	95% CI	OR	***p***-value^**b**^	95% CI
miR-17-5p	0.94	0.90	0.32; 2.76	0.79	0.68	0.26; 2.39
miR-134-5p	1.04	0.82	0.73; 1.48	1.02	0.91	0.71; 1.48
miR-144-5p	0.76	0.13	0.53; 1.08	0.74	0.11	0.51; 1.07
let-7b-5p	0.33	0.03	0.12; 0.91	0.33	0.04	0.12; 0.93
let-7c-5p	0.78	0.29	0.49; 1.24	0.77	0.28	0.48; 1.24

*OR* odds ratio, *CI* confidence interval

^*^∆Ct = Ct_miR-425-5p_ – Ct_miR of interest_

^a^Logistic regression

^b^Logistic regression adjusted for age and BMI

**Fig. 2:**
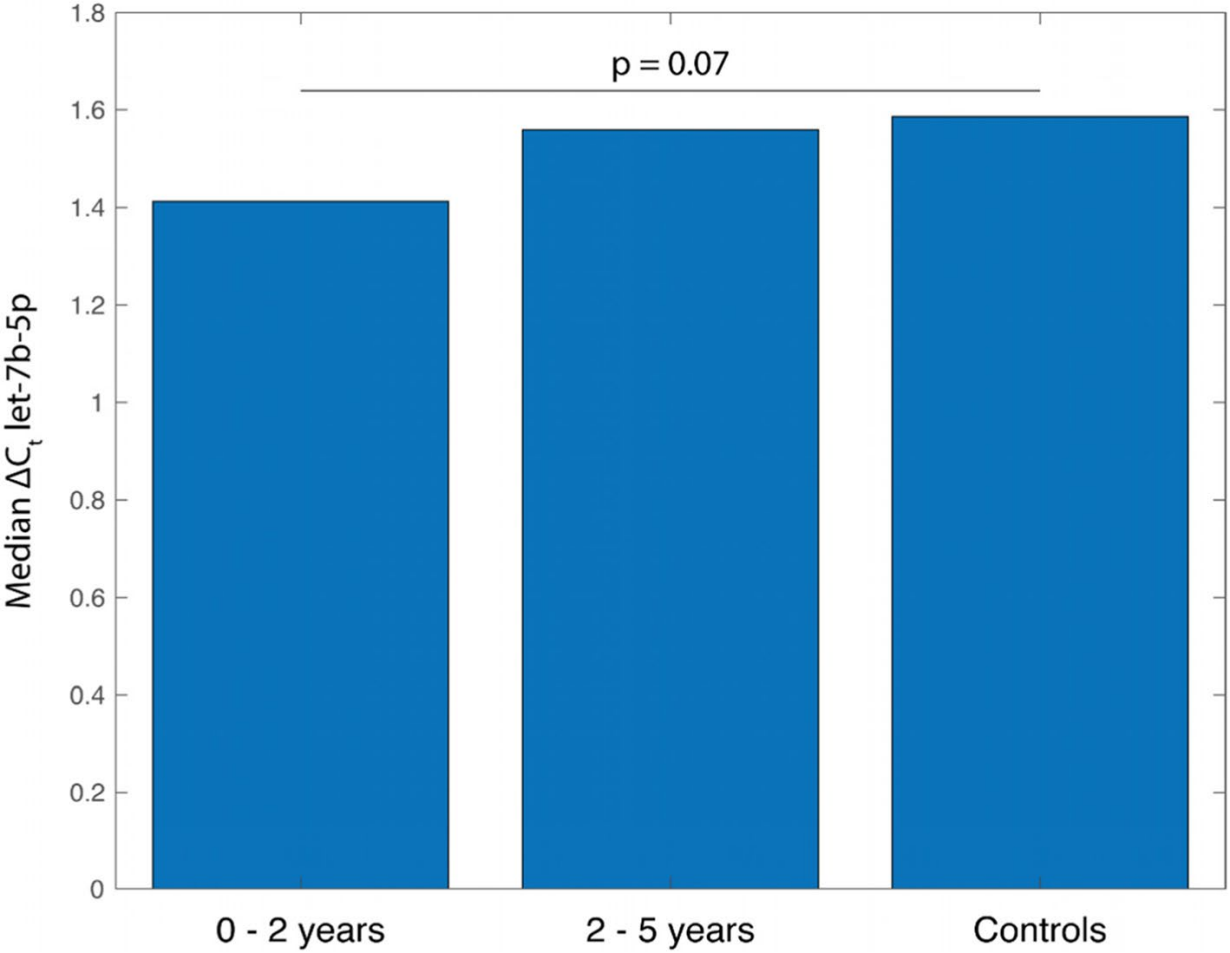
Median expression levels of let-7b-5p regarding time to MDD diagnosis during follow-up

Finally, the expression levels of the five miRNAs were analysed with respect to the time interval between the date of blood sampling and the date of MDD diagnosis. It showed that let-7b-5p exhibited a trend over time (Fig. 2), with larger differences in let-7b-5p levels between cases and controls for the earlier cases (MDD diagnosis <2 years from blood sampling) than later cases (MDD diagnosis 2–5 years from blood sampling). The observed trend approached borderline significance (*p* = 0.07).

## Discussion

To our knowledge, this is the first study investigating the association of circulating miRNA and the risk of MDD development. The main finding of this study was that let-7b-5p expression levels at baseline were significantly lower in plasma of individuals, who later developed MDD, compared to those who did not develop MDD. This suggests that let-7b-5p levels have potential as a circulating predictive biomarker in the risk assessment of the development of MDD. Other selected miRNAs had no significant difference in expression levels between cases (MDD) and controls (non-MDD).

Many major cellular functions such as development, differentiation, growth, and metabolism are known to be regulated by miRNAs. As miRNAs play a major role in developmental processes, dysregulation of miRNA has been shown to be responsible for the development of numerous diseases such as cancer, cardiovascular disease and neurodevelopmental disease. Since the discovery of circulating miRNAs, most researchers have reported the use of miRNAs as circulating biomarkers for the diagnosis or prognosis of diseases [23]. More recently, studies have identified biomarkers for risk prediction and early diagnosis of acute myocardial infarction [9, 24].

MiRNAs have dynamic roles in neurogenesis [25]. Therefore, it is not surprising that they were found to play a major role in neurodegenerative diseases. MiRNAs were differentially expressed in the brains of patients with Alzheimer’s disease [26]. In addition, dysregulation of miRNA in mice brain resulted in a phenotype similar to Parkinson’s disease [27].

MiRNA levels in peripheral blood reflect the miRNA levels in the central nervous system (CNS) as miRNA and other circulating nucleic acids can cross the blood-brain barrier (BBB) under physiological conditions [28]. Pathological conditions may disrupt the BBB, which further increases the permeability allowing for the open flow of molecules into the peripheral circulation. This indicates that peripheral blood reflects the level of miRNAs from the CNS [28]..

In the present study, let-7b-5p was found to be negatively associated with the risk of MDD development. Let-7b-5p is part of the let-7 family. The let-7 family of miRNAs was first identified as a regulator of developmental timing in *C. elegans* by Reinhart et al. [29]. Let-7 is highly conserved in animal species, but has multiple isoforms in higher species. In humans, nine mature isoforms have been identified; one of which is let-7b-5p [30].

Gururajan et al. [17] found a significant reduction in expression of let-7b-5p in patients with treatment-resistant depression (TRD) receiving electroconvulsive stimulation therapy (ECT) compared to controls. They also observed a trend toward higher post-treatment expression of let-7b-5p in patients who received ketamine treatment (KET) compared to those who received ECT. Inconsistent data were reported by Belzeaux. et al. [31] showed that let-7b-5p expression was upregulated in major depressive episodes. This analysis of let-7b-5p expression was based on peripheral blood mononuclear cells (PBMC) , which are not fully consistent with the miRNA changes in plasma used in the present study.

Let-7b-5p has been identified as a possible positive regulator of the extracellular signal-regulated kinase 1/2 (ERK1/2) signaling pathway [32]. ERK1/2 signaling is known to play a major role in synaptic and structural plasticity and has been associated with stress and depression. A study by Dwivedi and Zhang [33] examined the ERK1/2 signaling in the frontal cortex and hippocampus of rats showing vulnerability (learned helplessness (LH)) and rats showing resilience (non-learned helplessness (non-LH)) to the development of stress-induced depression. They determined both activation and expression of ERK1 and ERK2 at the transcriptional and translational level and found both protein and mRNA levels to be significantly decreased in hippocampus and frontal cortex of LH rats. These results suggest the involvement of ERK1/2 signaling in generating vulnerability to depression [33]. Thus, downregulation of let-7b-5p leads to a decrease in ERK1/2 signaling, which in turn is associated with an increased risk of MDD development.

This study is the first nested case-control study to explore potential biomarkers for the risk prediction of MDD development. The results of this study were promising, but the limitations of the study must be kept in mind when evaluating the results. Firstly, the sample size was relatively low. The study design, though, required a smaller minimum sample size than what was used in the study. Secondly, cases and controls were unmatched in the study design. This, however, was corrected via adjustments in age and BMI in the statistical analysis. In addition, family history is a major factor concerning the risk assessment of MDD development [34, 35] The effect of family history, however, could not be assessed as this confounder was not included in the WHILA cohort study [18]. Finally, our research indicates that let-7b-5p has the potential as a circulating predictive biomarker of MDD, but functional studies have to be performed in the future to confirm the potential role of miRNAs in MDD risk prediction.

## Conclusions

In summary, the findings of this study show that plasma levels of let-7b-5p have the potential as a biomarker of risk prediction for MDD development. Future studies are needed to validate and confirm the results in different cohorts with larger sample sizes.

## Supplementary Information

**Additional file 1: Table S1.** Correlation between peripheral blood biomarkers and let-7b-5p expression levels.

## Data Availability

The datasets generated during and/or analysed during the current study are not publicly available due to privacy concerns; please contact the corresponding author for more information.

## Abbreviations

MDD: Major depressive disorder
CVD: Cardiovascular disease
ncRNA: Non-coding RNA
miRNA: microRNA
mRNA: Messenger RNA
UTR: Untranslated region
CSF: Cerebrospinal fluid
HC: Healthy control
SCZ: Schizophrenia
BD: Bipolar disorder
CBT: Cognitive behavioural therapy
RCT: Randomised control trial
WHILA: Women’s Health in Lund Area
CPF: Center for Primary Health Care Research
ICD: International Classification of Diseases
Ct: Threshold cycle value
SD: Standard deviation
BMI: Body mass index
TG: Triglycerides
TC: Total cholesterol
HDL-C: High-density lipoprotein cholesterol
LDL-C: Low-density lipoprotein cholesterol
OR: Odds ratio
CI: Confidence interval
CNS: Central nervous system
BBB: Blood-brain barrier
TRD: Treatment-resistant depression
KET: Ketamine treatment
ECT: Electroconvulsive stimulation
PBMC: Peripheral blood mononuclear cells
ERK1/2: Extracellular signal-regulated kinase 1/2
LH: Learned helplessness
non-LH: Non-learned helplessness

## References

[1] Fava M, Kendler KS (2000). Major depressive disorder. Neuron..

[2] Depression and the global economic crisis: is there hope? Lancet. 2012;380(9849):1203. 10.1016/S0140-6736(12)61694-8. 10.1016/S0140-6736(12)61694-8 23040842

[3] Soleimani L, Lapidus KAB, Iosifescu DV (2011). Diagnosis and treatment of major depressive disorder. Neurol Clin.

[4] Kennis M, Gerritsen L, van Dalen M, Williams A, Cuijpers P, Bockting C (2020). Prospective biomarkers of major depressive disorder: a systematic review and meta-analysis. Mol Psychiatry.

[5] Lohoff FW (2010). Overview of the genetics of major depressive disorder. Curr Psychiatry Rep.

[6] Uchida S, Yamagata H, Seki T, Watanabe Y (2018). Epigenetic mechanisms of major depression: targeting neuronal plasticity. Psychiatry Clin Neurosci.

[7] Bartel DP (2004). MicroRNAs: genomics, biogenesis, mechanism, and function. Cell..

[8] Maffioletti E, Tardito D, Gennarelli M, Bocchio-Chiavetto L (2014). Micro spies from the brain to the periphery: new clues from studies on microRNAs in neuropsychiatric disorders. Front Cell Neurosci.

[9] Bye A, Røsjø H, Nauman J, Silva GJ, Follestad T, Omland T, Wisløff U (2016). Circulating microRNAs predict future fatal myocardial infarction in healthy individuals - the HUNT study. J Mol Cell Cardiol.

[10] Mitchell PS, Parkin RK, Kroh EM, Fritz BR, Wyman SK, Pogosova-Agadjanyan EL, Tewari M (2008). Circulating microRNAs as stable blood-based markers for cancer detection. PNAS..

[11] Salloum-Asfar S, Satheesh NJ, Abdulla SA. Circulating miRNAs, small but promising biomarkers for autism Spectrum disorder. Front Mol Neurosci. 2019;12(253). 10.3389/fnmol.2019.00253. 10.3389/fnmol.2019.00253 PMC6808050 31680857

[12] Wang X, Sundquist J, Zöller B, Memon AA, Palmér K, Sundquist K, Bennet L (2014). Determination of 14 circulating microRNAs in swedes and Iraqis with and without diabetes mellitus type 2. PLoS One.

[13] Smalheiser NR, Lugli G, Zhang H, Rizavi H, Cook EH, Dwivedi Y (2014). Expression of microRNAs and other small RNAs in prefrontal cortex in schizophrenia, bipolar disorder and depressed subjects. PLoS One.

[14] Camkurt MA, Acar Ş, Coşkun S, Güneş M, Güneş S, Yılmaz MF, Tamer L (2015). Comparison of plasma MicroRNA levels in drug naive, first episode depressed patients and healthy controls. J Psychiatr Res.

[15] Hp Z, Xl L, Jj C, Cheng K, Bai SJ, Zheng P, Xie P (2020). Circulating microRNA 134 sheds light on the diagnosis of major depressive disorder. Transl. Psychiatry..

[16] Wang X, Sundquist K, Hedelius A, Palmér K, Memon AA, Sundquist J (2015). Circulating microRNA-144-5p is associated with depressive disorders. Clin Epigenetics.

[17] Gururajan A, Naughton ME, Scott KA, O'Connor RM, Moloney G, Clarke G, Dinan TG (2016). MicroRNAs as biomarkers for major depression: a role for let-7b and let-7c. Transl Psychiatry.

[18] Samsioe G, Lidfeldt J, Nerbrand C, Nilsson P (2010). The Women's health in the Lund area (WHILA) study - an overview. Maturitas..

[19] Ding L, Gu H, Xiong X, Ao H, Cao J, Lin W, Cui Q (2019). MicroRNAs involved in carcinogenesis, prognosis, therapeutic resistance and applications in human triple-negative breast Cancer. Cells..

[20] Vasu S, Kumano K, Darden CM, Rahman I, Lawrence MC, Naziruddin B (2019). MicroRNA signatures as future biomarkers for diagnosis of diabetes states. Cells..

[21] Bao MH, Feng X, Zhang YW, Lou XY, Cheng Y, Zhou HH (2013). Let-7 in cardiovascular diseases, heart development and cardiovascular differentiation from stem cells. Int J Mol Sci.

[22] Blondal T, Jensby Nielsen S, Baker A, Andreasen D, Mouritzen P, Wrang Teilum M, Dahlsveen IK (2013). Assessing sample and miRNA profile quality in serum and plasma or other biofluids. Methods..

[23] Creemers EE, Tijsen AJ, Pinto YM, Rooij E (2012). Circulating MicroRNAs. Circ Res.

[24] Wang KJ, Zhao X, Liu YZ, Zeng QT, Mao XB, Li SN, Chen ZJ (2016). Circulating MiR-19b-3p, MiR-134-5p and MiR-186-5p are promising novel biomarkers for early diagnosis of acute myocardial infarction. Cell Physiol Biochem.

[25] Lang MF, Shi Y (2012). Dynamic roles of microRNAs in neurogenesis. Front Neurosci.

[26] Lukiw WJ (2007). Micro-RNA speciation in fetal, adult and Alzheimer's disease hippocampus. Neuroreport..

[27] Cuellar TL, Davis TH, Nelson PT, Loeb GB, Harfe BD, Ullian E, McManus MT (2008). Dicer loss in striatal neurons produces behavioral and neuroanatomical phenotypes in the absence of neurodegeneration. PNAS..

[28] Bruno DCF, Donatti A, Martin M, Almeida VS, Geraldis JC, Oliveira FS, Dogini DB, Lopes-Cendes I (2020). Circulating nucleic acids in the plasma and serum as potential biomarkers in neurological disorders. Braz J Med Biol Res.

[29] Reinhart BJ, Slack FJ, Basson M, Pasquinelli AE, Bettinger JC, Rougvie AE, Ruvkun G (2000). The 21-nucleotide let-7 RNA regulates developmental timing in Caenorhabditis elegans. Nature..

[30] Lee H, Han S, Kwon CS, Lee D (2016). Biogenesis and regulation of the let-7 miRNAs and their functional implications. Protein Cell.

[31] Belzeaux R, Bergon A, Jeanjean V, Loriod B, Formisano-Tréziny C, Verrier L, Ibrahim EC (2012). Responder and nonresponder patients exhibit different peripheral transcriptional signatures during major depressive episode. Transl Psychiatry.

[32] Ham O, Lee SY, Lee CY, Park JH, Lee J, Seo HH, Hwang KC (2015). Let-7b suppresses apoptosis and autophagy of human mesenchymal stem cells transplanted into ischemia/reperfusion injured heart 7by targeting caspase-3. Stem Cell Res Ther.

[33] Dwivedi Y, Zhang H. Altered ERK1/2 signaling in the brain of learned helpless rats: relevance in vulnerability to developing stress-induced depression. Neural Plast. 2016:7383724. 10.1155/2016/7383724. 10.1155/2016/7383724 PMC4709739 26839717

[34] Sullivan PF, Neale MC, Kendler KS (2000). Genetic epidemiology of major depression: review and meta-analysis. Am J Psychiatry.

[35] Zalar B, Blatnik A, Maver A, Klemenc-Ketiš Z, Peterlin B (2018). Family history as an important factor for stratifying participants in genetic studies of major depression. BJMG..

