# Supplementary material for: Association of circulating let-7b-5p with major depressive disorder: a nested case-control study
Source: BMC Psychiatry. 2021 Dec 9;21:616. doi: 10.1186/s12888-021-03621-4 (PMC8662878; doi:10.1186/s12888-021-03621-4)
Supplement: Supplementary file 1 — Additional file 1: Table S1. Correlation between peripheral blood biomarkers and let-7b-5p expression levels. [file 12888_2021_3621_MOESM1_ESM.docx]

**Supplementary data**

**Table S1:** Correlation between peripheral blood biomarkers and let-7b-5p expression levels

| Peripheral blood biomarker | p-value |
| --- | --- |
| Total cholesterol | 0.98 |
| Triglycerides | 0.97 |
| Glucose | 0.78 |
| HDL-C | 0.97 |
| LDL-C | 0.90 |
